# Early and accelerated access programs for medical devices in the European Union: mapping regulatory derogations and national schemes

**DOI:** 10.3389/fmedt.2026.1729631

**Published:** 2026-02-13

**Authors:** Baptiste Haon, Mira Hartmann, Sanae Akodad, Hilde Stevens, Lise Rochaix

**Affiliations:** 1 Hospinnomics, Paris School of Economics, Paris, France; 2 GATE UMR CNRS 5824 Université Lyon 2, Lyon, France; 3 Sciences Po, Paris, France; 4 Institute for Interdisciplinary Innovation in Healthcare (I3h), Faculté de Médecine, Solvay Brussels School of Economics and Management, Université Libre de Bruxelles (ULB), Brussels, Belgium

**Keywords:** accelerated access, Article 59, compassionate use, coverage with evidence development, early access, equity, medical devices, Regulation (EU) 2017/745

## Abstract

**Background:**

Early access programs (EAPs) can provide patients with medical devices before full market access in urgent or high unmet-need situations. Unlike pharmaceuticals, the European Union (EU) has no harmonised early-access framework for medical devices, resulting in fragmented national practices. We use *EAP* as an umbrella term for two overlapping categories with distinct objectives: (1) *early access in the strict sense*, authorising exceptional pre-market use under defined conditions; and (2) *accelerated access*, aiming to shorten time to routine access by streamlining regulatory, reimbursement, or evidence-generation pathways. We map and compare these mechanisms across the 27 EU Member States (MS).

**Methods:**

We conducted a qualitative document-based analysis of peer-reviewed literature, grey literature (policy papers, regulatory guidance), and legal sources (EU legislation, national laws) published up to June 2025. We searched PubMed and Google Scholar, consulted Eur-Lex, and reviewed national competent authority websites. Search terms (English and local languages, then translated) combined “medical device” with “early access,” “accelerated access,” “compassionate use,” and “derogation,” plus “EU” or MS names. Documents were included if they described mechanisms enabling early use of medical devices. We synthesised and compared key program characteristics.

**Results:**

We identified early and accelerated access pathways at EU level and within MS. EU law provides derogations from CE-marking requirements, notably Article 59 of the EU Medical Device Regulation (EU MDR), allowing national authorisation of non-CE-marked devices in exceptional circumstances. The EU MDR also enables the use of custom-made devices and certain in-house manufactured devices by health institutions without full CE marking. Nationally, at least half of MS operate compassionate or expanded access schemes for devices, and some implement reimbursement or evidence-development mechanisms (e.g., France’s *forfait innovation*) to support early use while generating decision-relevant data.

**Conclusions:**

Early access to medical devices in Europe relies on EU regulatory derogations and heterogeneous national schemes. The landscape remains fragmented and unevenly documented. We recommend: (1) a more structured EU-level route for high-risk and combination products, (2) lifecycle-based national schemes integrating regulatory and payer access, (3) measures to reduce inequities and administrative barriers, particularly in Central and Eastern Europe, and (4) improved transparency and standardised data collection on implemented pathways.

## 1 Introduction

In this article, we focus on the regulatory context of the European Union (EU) Member States [Member State (MS)]. Innovative medical devices can offer lifesaving or life-improving interventions, but obtaining full market authorization [Conformité Européenne (EU conformity marking) (CE)-marking in the EU under the MDR [15]] requires completion of conformity assessment procedures and clinical evaluations. This process can take years, delaying patient access to novel technologies. In the pharmaceutical sector, most EU MS and the European Medicines Agency (EMA) have well-established early access mechanisms (e.g., compassionate use programs) to provide treatments to patients with serious conditions before marketing authorization [1, 10, 11]. By contrast, for medical devices, the regulatory framework has historically been decentralized and less explicit regarding pre-market access. Approaches to pre-market or early access can differ substantially across jurisdictions.

Under the previous EU framework—Medical Devices Directive (93/42/EEC) (MDD) Council Directive 93/42/European Economic Community (EEC) and Council Directive 90/385/EEC [Active Implantable Medical Device Directive (90/385/EEC) (AIMDD)]—the requirements for placing devices on the market differed from the current European Union Medical Device Regulation [Regulation (EU) 2017/745] (EU-MDR), which repealed those directives and strengthened clinical evaluation and post-market obligations (2, 15, 45). However, there was no EU-wide, harmonised scheme for compassionate or expanded access to non-CE-marked medical devices; early access was managed largely at the national level, and approaches varied widely.

In this paper, “market access” refers to regulatory access (CE-marking and lawful placing on the market/putting into service) and/or payer access (coverage and reimbursement). We use “early access” for exceptional pre-CE use in patients, and “accelerated access” for tools that shorten regulatory, evidence, or reimbursement timelines.

Currently, two mechanisms for prompt access to medical devices are used across EU MSs: “early access” and “accelerated access.” Both refer to any program, framework, or regulatory pathway that enables patients to be treated with a medical device before its full CE-marking approval and/or before routine payer coverage. The important difference between these two scheme types is that Early Access Program(s) (EAP) in the strict sense allow exceptional access to a medical product under specific conditions, and Accelerated Access Program(s) (AAP) aim to reduce the time to market authorization of a medical product by streamlining conformity assessment itself, clinical evaluation, evidence generation, or reimbursement negotiations.

The categories overlap because some schemes combine exceptional clinical use with accelerated evidence generation or conditional coverage; however, their primary objective differs (patient access under exception vs shortening pathways to routine market/coverage).

We classify a mechanism as EAP when it authorises patient use of a non-CE-marked device under exceptional conditions. We classify a mechanism as AAP when it accelerates time to CE-marking and/or reimbursement through prioritised assessment, streamlined evidence pathways, or conditional coverage.

We first describe EU-level derogations and guidance under MDR/IVDR, then map national mechanisms, distinguishing (i) exceptional pre-CE use, (ii) emergency derogations, and (iii) conditional reimbursement/coverage schemes.

This review focuses on medical devices. Under Union law, a “medical device” is defined in Article 2(1) of Regulation (EU) 2017/745 {Medical Devices Regulation [Regulation (EU) 2017/745] (MDR)} as any instrument, apparatus, appliance, software, implant, reagent, material or other article intended by the manufacturer to be used for one or more specific medical purposes. By definition, the principal intended action of a medical device is not achieved by pharmacological, immunological, or metabolic means, although it may be assisted by such means [15].

This definition includes a broad spectrum of products, each with distinct features and varying levels of health risk. The MDR classifies medical devices into four risk-based categories (here listed with illustrative examples; for the full picture please refer to the MDR) [15]:

1. **Class I:** Low-risk, non-invasive devices (e.g., wheelchairs, crutches, glasses, surgical masks, bandages).
2. **Class IIa:** Low to medium-risk devices, typically used in the short term (between 60 min and 30 days) (e.g., diagnostic ultrasound devices, hearing aids, contact lenses, dental fillings).
3. **Class IIb:** Medium to high-risk devices, often used long-term (more than 30 days) (e.g., dental implants, defibrillators, respiration and dialysis devices).
4. **Class III:** High-risk devices (e.g., coronary stents, heart valves, endoprostheses, absorbable surgical sutures).

While many devices require notified body involvement, Class I devices (non-sterile, non-measuring, without a reusable surgical instrument function) may be placed on the market under manufacturer self-certification, whereas higher-risk devices (Classes IIa, IIb, III) require conformity assessment with the involvement of a notified body. Assessment requirements for CE-marking are stricter for higher risk devices than for lower risk devices [15].

Particularly for Class IIb and Class III medical devices, having functional EAP and AAP is critical. This is because higher-risk devices typically face more demanding clinical evidence expectations and more complex conformity assessment procedures, which can lengthen time-to-market; when such devices target serious conditions, delays may translate into substantial forgone health benefits for patients. Both are particularly important in two cases: first, when a medical device represents a “major clinical benefit vs state of the art”; second, when a treatment addresses an unmet medical need [32]. Devices in Classes IIb and III are often used in higher-acuity clinical settings, where claims of unmet medical need and potential major clinical benefit are more likely to be central to the access decision. However, MDR risk class and “breakthrough”^1^ status are not mechanically linked and depend on the specific indication and the state of the art [32].

Therefore, this article focuses more strongly on schemes that address higher-risk medical devices under the EU MDR, in particular Class IIb and Class III devices, and considers fewer schemes targeted at Class I and IIa medical devices. This prioritisation reflects the fact that higher-risk classes typically face more stringent evidence and conformity-assessment requirements, and thus are more exposed to access delays. For instance, we do not provide an exhaustive mapping of stand-alone medical device software/software-based medical devices (SaMD/MD software). This is not because such products are limited to lower MDR classes—under MDR Rule 11, they may fall into Class IIa, IIb, or III depending on intended purpose and clinical impact—but because software-enabled devices raise distinct governance issues (e.g., frequent updates, change control, and interaction with the AI Act) that warrant a dedicated treatment. However, there may be exceptions. For example, in health emergencies, EAP for Class I and IIa devices may also be essential, as experienced in the COVID-19 pandemic. The case of EAP and AAP in health emergencies will also be discussed. While this review emphasizes high-risk and combination devices, digital medical devices (including Artificial Intelligence-enabled software) increasingly raise early/accelerated access questions. Some Class IIa software-based devices may qualify as breakthrough technologies, and AI systems, especially those updated iteratively, create decision uncertainty that evolves over time. We therefore discuss digital/AI devices as a cross-cutting case for lifecycle governance, evidence generation, and risk allocation.

Throughout, we interpret early and accelerated access as decision-making under evidential uncertainty with learning over time. Early access can bring expected health gains forward in time for patients who would otherwise wait for routine regulatory and payer decisions. At the same time, earlier use typically occurs under greater evidential uncertainty, which increases residual clinical risk for patients and users. In parallel, early procurement or coverage decisions can shift financial risk onto hospitals and payers unless explicit safeguards and risk-sharing arrangements are in place.

From a medical device perspective, the central aim is to support efficient decisions about adoption and reimbursement under uncertainty. The concepts below help operationalise this aim under uncertainty. Three economic concepts organise the trade-offs: (i) *option value of learning-by-using*—early, protocolised use can generate information that improves future decisions; (ii) *risk sharing*—contract terms and coverage rules can reallocate downside risk when evidence is thin; and (iii) *dynamic incentives*—policy design today affects firms’ future investment in clinically relevant evidence and truly novel devices. We therefore assess schemes not only by legal basis but by whether they internalise information externalities, i.e., whether early use is organised to (i) generate decision-relevant evidence that is consolidated and fed back into regulatory, clinical, and payer decisions (rather than remaining fragmented across actors), (ii) allocate clinical and financial risk transparently and efficiently, and (iii) preserve incentives for innovation while remaining compatible with budget constraints.

Today, medical devices are increasingly available in combination with medicinal products. Indeed, it is not always possible to make a clear-cut distinction between the types of products for regulatory purposes. For instance, drug–device combination products (e.g., device components incorporating, as an integral part, a substance that, if used separately, would be considered a medicinal product) raise specific regulatory questions because the conformity assessment requires input from medicines authorities. Accordingly, we treat device-drug combinations as a *special regulatory interface case* in this review (rather than as an example of pre-market early access *per se*), because early or accelerated access decisions for such products may hinge on both device evidence requirements and the additional medicinal-substance assessment steps.

The objective of this study is to review formal legal instruments, non-binding policies and guidance documents that shape early and accelerated *regulatory pathways* (covering both EAP and AAP as defined above) for medical devices in the EU and in EU MS. Moreover, beyond identifying all existing programs, the review uses a scoping-review approach to compare their features and requirements and critically analyse strengths, limitations and gaps, particularly when comparing these mechanisms for medical devices with the more established EAP infrastructure of medicinal products.

## 2 Methodology

This study adopts a document-based policy analysis to examine early and accelerated access mechanisms for medical devices in the European Union. The objective is not to assess clinical effectiveness, but to map regulatory and payer-facing pathways, identify their legal bases and design features, and analyse how they address uncertainty, evidence generation, and access constraints for innovative medical devices. The focus is explicitly on EAP and AAP, as defined in the introduction, across EU-level frameworks and national implementations.

### 2.1 Study design and scope

We conducted a qualitative analysis of legislative texts, regulatory guidance, policy documents, and selected academic literature published or in force between 2010 and June 2025. The temporal scope reflects the progressive transition from the former medical device directives to the MDR/In Vitro Diagnostic Medical Devices Regulation [Regulation (EU) 2017/746] (IVDR) framework and the subsequent emergence of national early-access and accelerated-access schemes. The geographic scope covers the EU as a whole, with specific attention to national approaches implemented by individual MSs.

### 2.2 Data sources

Three categories of sources were examined: (i) *EU-level legal and regulatory sources*, including Regulation (EU) 2017/745 and 2017/746, delegated and implementing acts, and non-binding but authoritative Medical Device Coordination Group (MDCG) guidance documents; (ii) *national-level documents*, including laws, decrees, and official guidance issued by competent authorities, HTA bodies, or payers describing early use, derogations, compassionate access, or conditional reimbursement schemes for medical devices; and (iii) *secondary sources*, including peer-reviewed articles, policy reports, and institutional analyses that describe or evaluate early or accelerated access mechanisms.

### 2.3 Search strategy

Searches were conducted between March and June 2025 in PubMed and Scopus for peer-reviewed literature, and through targeted searches of institutional websites (European Commission, MDCG repository, national competent authorities, HTA bodies, and payer institutions). Search terms combined device-related concepts (“medical device,” “in vitro diagnostic,” “software as a medical device,” “medical device software”) with access-related concepts (“early access,” “compassionate use,” “expanded access,” “Article 59,” “derogation,” “conditional coverage,” “coverage with evidence development,” “fast track”). Searches were restricted to documents relating to the EU or individual MSs. Reference lists of key documents were screened iteratively to identify additional relevant sources.

### 2.4 Inclusion and exclusion criteria

Documents were included if they described a formal or de facto mechanism enabling (i) the use of non-CE-marked medical devices in patients, or (ii) accelerated regulatory, evidentiary, or reimbursement pathways for devices prior to routine market or payer access. We excluded documents that addressed only pharmaceuticals, general innovation policy without device-specific mechanisms, or purely technical standards without access implications.

### 2.5 Data extraction and analytical framework

For each identified mechanism, we extracted descriptive attributes including: legal basis, governance level (EU or national), target device categories, eligibility criteria, evidence requirements, duration and scope of access, and links to reimbursement or post-market obligations. These features are summarised in an operational typology (Table 1).

**Table 1:** Operational typology and key extracted features for early and accelerated access mechanisms.

Category	Level	Primary entry point	Typical legal basis	Key extracted features (examples)
EAP (exceptional pre-CE use)	EU/national	Competent authority/hospital	MDR Art. 59; national exceptional-use provisions	Eligibility (serious/unmet need), authorization scope (named-patient/cohort), consent, reporting, time/volume limits
Emergency derogations	EU/national	Competent authority (crisis)	MDR Art. 59 in crisis; emergency decrees	Broader population scope, expedited documentation, post-hoc traceability, transition to conformity
AAP (accelerated pathway to routine access)	National	Payer/HTA (often with regulator)	Conditional coverage, CED, innovation funds, fast-tracks	Time-limited reimbursement, study obligations, renewal/exit rules, price/risk-sharing clauses

Mechanisms are classified by their primary objective. EAP authorise patient use of a non-CE-marked device under exception; AAP shorten time to routine access by accelerating assessment and/or reimbursement. Hybrid schemes are tagged by a secondary category where applicable (e.g., EAP→AAP).

Beyond descriptive mapping, the analysis is structured around three economic and policy concepts that inform the interpretation of early and accelerated access schemes: (i) information production through learning-by-using, (ii) management and sharing of clinical and financial risk under uncertainty, and (iii) dynamic incentives for evidence generation and innovation. These concepts are not used as formal evaluation metrics but as an interpretive lens in the Results and Discussion sections to compare approaches across MSs and to identify design trade-offs and policy gaps.

### 2.6 Methodological positioning

While the study draws on elements commonly used in scoping reviews, it does not aim to provide an exhaustive inventory of all documents nor a systematic appraisal of evidence quality. Instead, it should be understood as a structured policy analysis that integrates legal, regulatory, and economic perspectives to characterise the current EU landscape of early and accelerated access for medical devices and to inform policy recommendations.

## 3 Regulatory pathways for early device access in the EU

This section maps the EU-level legal instruments and influential guidance that can enable exceptional pre-CE use (early access) or shorten time to routine access (accelerated access). We first summarise EU-level derogations, exemptions, and special interface cases under the MDR/IVDR, before turning to national schemes that operationalise early and accelerated access in practice.

### 3.1 EU-level framework

#### 3.1.1 Derogative early access in the medical device regulation

At the EU level, the primary legal framework is Regulation (EU) 2017/745 (EU-MDR) [15], which fully replaced the earlier medical device directives in May 2021. An overview of the EU-level legal provisions and guidance documents cited in this section is provided in Supplementary Appendix Tables 2, 3. The EU-MDR sets the general rule that, to be placed on the market or put into service in the EU, a medical device must carry CE-marking, indicating conformity with all regulatory requirements. The CE-marking is provided following conformity assessment involving notified bodies designated by MS authorities and notified under Union procedures [24]. After receiving CE-marking, manufacturers can market their product in any EU MS.

The EU-MDR includes provisions that can facilitate early access to medical devices in certain situations:

##### 3.1.1.1 National derogation (EU-MDR Article 59)

Article 59 EU-MDR allows a MS competent authority to authorize, on a duly justified request, the placing on the market and putting into service within its territory of a specific device for which the conformity assessment procedures have not been carried out, in the interest of public health or patient safety or health [28]. Such authorization is typically reserved for exceptional circumstances. The MS must inform the European Commission (EC) and other MSs; the Commission can assess whether the derogation should be extended EU-wide.

##### 3.1.1.2 Custom-made devices

A custom-made device is one specifically made per a healthcare professional’s prescription to meet the unique needs of an individual patient. Such devices do not require CE-marking and can be supplied for that patient without a full conformity assessment [15].

However, the manufacturer must still meet general safety and performance requirements and document the device’s design and intended use. This provision, carried over into the EU-MDR 2017/745, enables patients to receive bespoke solutions when no off-the-shelf device would be suitable. It effectively provides access to devices that, by their nature, will not undergo conventional market approval. The trade-off is that oversight is limited to post-hoc registration and the clinician’s responsibility, rather than pre-market review.

##### 3.1.1.3 In-house manufacturing by health institutions [EU-MDR Article 5(5)]

The EU-MDR allows health institutions to manufacture and use certain devices internally, without CE-marking, under specific conditions (quality management, documentation, and justification that no equivalent CE-marked device is available to meet the target patient group’s needs) [15, 31].

Notably, unlike for medicines, there is no centralized EU EAP program administered by EMA or another body for medical devices. EU-level provisions take the form of legal exceptions rather than structured programs. This is also reflected in the centrality of MS to these derogations. For instance, EU-MDR 2017/745 Art. 59 requires action by individual MS and is typically reserved for exceptional circumstances (e.g., public health emergencies or compassionate cases where the patient’s life is at stake). In terms of nonbinding guidance, the EC and the MDCG have issued some guidance documents to clarify the application of these provisions. For example, in 2020, the MDCG issued guidance on the Article 59 procedure to encourage coordination during COVID-19, outlining the criteria and documentation needed for a derogation [7, 38]. However, such guidance does not replace national decision making and is not a formal program.

#### 3.1.2 The exception for orphan medical devices

Although orphan devices are not the primary scope of this review (they are treated in a companion paper by two of the authors), we briefly summarise EU guidance because it shapes the boundaries of feasible evidence generation and interacts with current and proposed acceleration tools.

The EU-MDR introduced greater safety requirements compared to the previous framework [26]. These increased evidence requirements can be challenging for orphan medical devices, which the MDCG defines as devices targeting conditions affecting no more than 12,000 patients per year in the EU and for which available alternatives are insufficient or the device represents a clinically more effective treatment [15]. To ensure access to orphan medical devices, despite the EU-MDR 2017/745, the MDCG composed of representatives of the Member States and chaired by the EC in 2024 published guidance on the clinical evaluation of orphan devices [29]. In particular, the guidance allows limitations in the provision of pre-market clinical data for orphan medical devices under the condition that [29]:

- All available non-clinical and clinical data has been evaluated, and that any limitations have been identified;
- Existing non-clinical and limited clinical data is sufficient to demonstrate that the relevant General Safety and Performance Requirements (GSPRs in Annex I MDR) are met, that the benefit-risk ratio is acceptable, and that it is expected that the device will provide a clinical benefit taking into account the clinical condition, the state of the art, and the safety of patients;
- Generating more pre-market data is not feasible or proportional within an acceptable time frame;
- There must be a robust post-market clinical follow-up plan (PMCF) that ensures clinical data generation after market authorization;
- Users are clearly informed of the orphan status and data limitations.

The guidance points to further flexibilities by the MDR to allow earlier market approval of orphan medical devices with emphasis on the use of non-clinical and real-world data, use of off-label evidence, and more flexible clinical trial requirements for high-risk medical devices [29].

In this sense, the guidance does not introduce a new legal tool to accelerate access to orphan medical devices but offers explanations and argumentation to manufacturers and notified bodies on how to use the leeway that the EU-MDR 2017/745 provides to facilitate access to orphan medical devices. Hence, there is no legal obligation for notified bodies to follow the guidance in the approval of medical devices. Nevertheless, the MDCG guideline offers a document that can be referred to when interpreting the EU-MDR 2017/745 [29].

#### 3.1.3 The special case of device-drug combinations

As mentioned in the introduction, medical devices are also increasingly combined with pharmaceuticals: In this case, among others, EU-MDR 2017/745 Article 117 obliges notified bodies to seek the scientific opinion of an expert panel under the Clinical Evaluation Consultation Procedure (CECP) [15]. In particular, this is necessary for [12, 15]

1. Medicines used in combination with a medical device;
2. Medical devices with an ancillary medicinal substance;
3. Companion diagnostics;
4. Medical devices made of substances that are systematically absorbed;
5. High-risk medical devices.

In these cases, the EMA expert panel evaluates the Clinical Evaluation Assessment Reports issued by the relevant notified body and issues a scientific opinion within 60 days of submission of the request by the notified body [42]. There is no legal obligation to accept the scientific opinion by the EMA. However, in case of refusal, the EU-MDR 2017/745 requires notified bodies to justify such action in the final conformity assessment [42].

There are no specific early access schemes for these products. Instead, access depends on whether the product is classified as a medicinal product or a medical device and then enters the respective EAP and accelerated access programs. Whether a combination is classified as a device or a drug depends on which component is the principal [12]. For example, insulin pens are classified as medicines with an ancillary device, whereas drug-eluting stents are medical devices with the stent being the principal. Hence, combination devices with the medical device as the principal component are subject to the EU-MDR 2017/745 as well as EAP and accelerated access schemes for medical devices.

Nevertheless, EU drug–device combination products often face longer time-to-market because they must comply with both the medicinal-product authorisation framework and additional device-specific requirements (notably the MDR Article 117 notified-body assessment/opinion for integral drug–device combinations), adding an extra procedural layer alongside the competent authority review (EMA or national competent authorities, depending on the procedure) [9, 13, 15, 36]. This extended, multi-stakeholder review can increase the value of early-access or other accelerated-access pathways where available [9].

Device-drug combinations, classified as medical devices, do not have any additional early access scheme that may mitigate the double regulatory burden imposed on these, resulting from a clear division of market authorization of medicines and medical devices within the EU. Unlike the US, that offers a formal designation via the Humanitarian Use Device (HUD) program, there are no accelerated access programs for medical devices available at the EU level [3]. This mechanism concentrates more strongly on the national arena, as will be discussed subsequently.

#### 3.1.4 Digital medical devices and AI-enabled software: accelerated access beyond device–drug combinations

Digital medical devices—including stand-alone software and Software as a Medical Device (SaMD)—raise early and accelerated access questions that are not fully captured by the traditional focus on device–drug combinations. Under Union law, software can qualify as a medical device where it is intended by the manufacturer for a medical purpose and therefore falls within the scope of Regulation (EU) 2017/745 (MDR) or Regulation (EU) 2017/746 (IVDR) [14, 15]. In practice, qualification and risk classification for Medical Device Software (MDSW) are operationalised through MDCG guidance, notably MDCG 2019-11 (rev. 1, June 2025) [30]. Clinical evidence expectations for MDSW are further specified in MDCG 2020-1 [28].

A second regulatory layer now applies to a substantial subset of digital devices: the Artificial Intelligence Act [Regulation (EU) 2024/1689] (AIA), Regulation (EU) 2024/1689 [16]. AI-enabled medical devices are frequently treated as *high-risk AI systems* according to the AIA classification scheme, because they are safety components of products subject to third-party conformity assessment under Union harmonisation law (including MDR/IVDR). The MDCG, together with the Artificial Intelligence Board (AIB), published an FAQ (MDCG 2025-6, June 2025) clarifying the interplay between MDR/IVDR and the AIA [31].

A key challenge for AI-enabled software is not only initial market entry but the governance of post-market updates across successive software releases. Software can be updated frequently; for machine-learning-enabled products, updates may affect calibration, subgroup validity, and failure modes. Accordingly, a substantial part of the “acceleration” problem concerns *change management* after the initial CE-marking rather than exceptional pre-market access. In this context, predetermined change control planning (PCCP) is increasingly discussed as a way to pre-specify the scope of expected modifications and the associated verification/validation activities within the conformity assessment framework; EU guidance on MDR/IVDR–AI Act interplay explicitly points to this direction [31]. This increases the welfare stakes of post-market governance and supports an “agile regulation” perspective: explicit learning obligations and feedback loops from post-market monitoring into regulatory and payer decisions, rather than relying primarily on time-limited authorisations [34].

#### 3.1.5 EU framework recap: from Article 59 derogations to special cases

Summarising, at the EU level, there is no centralized Early Access Program (EAP) or Accelerated Access Program (AAP) for medical devices; instead, early access relies on legal derogations and flexibilities under Regulation (EU) 2017/745, notably: (1) Article 59 national derogations allowing temporary use of non-CE-marked devices in exceptional circumstances; (2) custom-made and in-house manufacturing exemptions that provide patient-specific access outside formal market approval; (3) MDCG guidance for orphan devices enabling flexible pre-market evidence requirements with reinforced post-market follow-up; and (4) for device–drug combinations, regulatory pathways involve dual assessment by notified bodies and European Medicines Agency without dedicated EAP/AAP schemes, often resulting in longer timelines. Finally, the regulatory baseline itself is evolving: on 16 December 2025, the European Commission published a proposal to amend MDR/IVDR that would introduce, inter alia, a new Article 52a on conformity assessment modalities for designated breakthrough devices (and orphan devices), signaling an intent to develop more explicit acceleration tools within the core medical device framework [8].

### 3.2 National schemes

EU MSs have developed their approaches, within the bounds of EU law, to allow early access to medical devices for patients in need. These include compassionate use programs, emergency derogations, and early innovative payment/evidence-generation schemes.

#### 3.2.1 Named-patient and cohort compassionate use programs

In several countries, physicians can request access to an unauthorized medical device for an individual patient or a defined group outside of a clinical trial [43]. For example, France has a well-defined process for “exceptional use” via Agence nationale de sécurité du médicament et des produits de santé (French National Agency for Medicines and Health Products Safety) (ANSM) [19]. Italy amended its national medical device law (Legislative Decree 46/1997) to allow early use for compassionate reasons [39]. Belgium provides a standard application form for compassionate use of a non-CE-marked device [17].

Germany relies on case-by-case permissions by Bundesinstitut für Arzneimittel und Medizinprodukte (Federal Institute for Drugs and Medical Devices, Germany) (BfArM), typically under Article 59 of the EU-MDR [35]. Similar reliance on Article 59 is common in Central and Eastern European [Central and Eastern Europe (CEE)] MSs. For example, Croatia’s Medical Devices Act explicitly empowers the Ministry of Health to approve use under Article 59 [40]. Slovenia’s Agency for Medicinal Products and Medical Devices of Slovenia (JAZMP) has published procedures for handling requests under Article 59 [25].

#### 3.2.2 Emergency or public health crisis authorizations

Many MSs can authorise non-CE-marked devices broadly during health emergencies. The COVID-19 pandemic was a key stress-test [38]. Countries issued emergency decrees or utilised existing laws to fast-track access to ventilators, Personal Protective Equipment (PPE), and diagnostic tests [7].

#### 3.2.3 Early innovative payment and evaluation schemes

Some countries link early access with evidence generation and reimbursement tools. France’s Forfait Innovation (French innovation funding scheme) (FI) provides temporary reimbursement while additional clinical evidence is collected [27, 33, 37]. France also implemented Prise En Charge Transitoire (French transitional coverage) (PECT) to bridge post-CE-marking access before reimbursement decisions [23]. Germany’s Neue Untersuchungs- und Behandlungsmethoden (new examination and treatment methods, Germany) (NUB) bridges reimbursement integration after CE-marking [5].

AAP can operationalize reimbursement fast-tracks for digital and AI-enabled health technologies by combining early, conditional coverage with structured evidence generation. In Germany, the Digitale Gesundheitsanwendung (Digital Health Application) (DiGA) Fast-Track—administered by BfArM—assesses eligible digital medical devices within a legally defined three-month review window, with the possibility of provisional listing during a time-limited trial phase [18, 41]. In France, Prise en charge anticipée numérique (Digital Advance Coverage) (PECAN) offers a derogatory, one-year reimbursement mechanism for sufficiently mature digital medical devices, enabling earlier patient access while benefit evidence is finalized and transitioned to common-law reimbursement [21, 22].

## 4 Discussion

Cross-country variation reflects alternative responses to common design problems. First, where early use is authorized without explicit obligations for structured data collection and transparent renewal criteria, evidence generation tends to be insufficient and poorly transferable across providers and payers. Second, ad hoc derogations frequently concentrate clinical and budgetary downside risk on clinicians and hospitals, whereas transitional coverage schemes with defined eligibility criteria and volume limits can distribute risk more efficiently. Third, predictable, lifecycle-based pathways—illustrated by the French case—can strengthen dynamic incentives by reducing policy uncertainty and encouraging investment in decision-relevant evidence.

Beyond device–drug combinations, “double regulation” increasingly characterises digital medical devices and, in particular, Artificial Intelligence (AI)-enabled MDSW. Where such software falls within the scope of MDR/IVDR, a substantial subset will also be subject to the AIA. For AI-enabled devices the relevant uncertainty is not only limited pre-market evidence but also *endogenous and dynamic*: post-deployment updates, model drift and context dependence can alter performance over time. This makes post-market monitoring and “data by design” central to any defensible acceleration strategy.

Our evidence base reflects the state of Union and national frameworks identified through June 2025. Since then, the European Commission has tabled a legislative proposal to amend both the MDR and the IVDR. The proposal (COM(2025) 1023 final) introduces, inter alia, a new Article 52a for designated *breakthrough devices* and *orphan devices*, signalling an intent to create prioritised assessment tools within the core framework rather than relying predominantly on discretionary derogations [8].

Based on this analysis, several key challenges emerge:

1. **Lack of EU-level early and accelerated access pathways for medical devices**
  The EU-MDR introduced a common denominator (Article 59 for derogations), but its use is still nationally driven. As a result, fragmentation can lead to inequitable access for patients. Several EU countries published a joint paper in the Council calling for a more centralized approach [20].
2. **Limited legal embedding of national early access tools for medical devices**
  Many MSs lack specific legal provisions for early access to medical devices and rely on ad hoc use of Article 59. France stands out via FI and PECT as transparent and predictable schemes [23].
3. **CEE MSs face gaps in the implementation of early access tools**
  Evidence and formal frameworks remain limited in CEE MSs. Administrative capacity constraints may reduce the feasibility of sophisticated schemes [6]. Regional cooperation (e.g., Benelux–Austria Initiative for cross-country cooperation in health (BeNeLuxA)) can support joint procurement and shared evidence generation [4].
4. **Need for a comprehensive, lifecycle-based approach to early and accelerated access**
  Early/accelerated access is relevant at two stages: before CE-marking (derogations/exceptional use) and after CE-marking but before payer coverage. Lifecycle approaches integrate both regulatory and reimbursement elements.
5. **Increase transparency on outcomes of early and accelerated access programs for medical devices**
  Limited transparency reduces utilisation and slows learning. Standardised reporting and anonymised sharing of outcomes would improve knowledge spillovers and safety monitoring.

## 5 Actionnable recommendations

These recommendations rest on three ideas: structured learning-by-using, reasoned risk sharing, and dynamic incentives. The goal is earlier access that is safe, fair, and worth it. To this end, the following recommendations are made:

### 5.1 An EU route for high-risk and combination products

At EU level, Article 59 has been an emergency valve rather than a pathway. It should be recast into a structured route for high–risk devices and device–drug combinations, with time limits, scope restrictions, Unique Device Identification (UDI)-based traceability, and explicit learning milestones.

### 5.2 Lifecycle national models

Member States need a lifecycle view in terms of reimbursement integration. Regulatory permission to place a device on the market or to use it under a derogation can be an end in itself, and many devices are used without specific reimbursement. However, for higher-cost technologies and for system-wide uptake, *effective patient access* typically requires both (i) legal authorisation for use (regulatory access) and (ii) a feasible financing route (payer access), whether through DRG/tariff mechanisms, procurement budgets, or explicit coverage decisions. Transitional coverage with evidence obligations can bound exposure and enable learning.

### 5.3 Closing the CEE gap

A small EU-funded support unit could provide templates and study designs; regional cooperation could lower costs and standardize methods.

### 5.4 Data by design

For early-access uses, Real-World Evidence (RWE) should follow existing frameworks [ISO 14155, ISO/TR 20416, IMDRF guidances; Post-Market Clinical Follow-up (PMCF) templates].

For digital devices and AI-enabled MDSW, “data by design” should explicitly incorporate *change control* and lifecycle evidence generation, because performance may evolve with software updates. This includes versioning and traceability, pre-specified criteria for when updates require re-validation and/or notified body involvement, staged roll-out where appropriate, and pre-defined stopping or rollback rules. In regulatory terms, these elements align with emerging predetermined change control planning (PCCP) approaches discussed in EU guidance on MDR/IVDR–AI Act interplay (MDCG 2025-6) and in parallel international work [e.g., FDA guidance [44]; forthcoming IMDRF guidance]. Where accelerated or conditional access is granted, continuous performance monitoring should be embedded within Post-Market Surveillance (PMS)/PMCF, including drift and subgroup surveillance, data-quality indicators, and escalation pathways.

### 5.5 Early dialogue and concurrency

Early multi-stakeholder scientific advice and evidence-plan alignment across regulators/competent authorities, notified bodies (within their remits), EMA where relevant, and Health Technology Assessment (HTA) bodies should align endpoints and evidence plans and reduce duplicated studies.

### 5.6 Roadmap and accountability

Progress should be visible via timelines, uptake across MSs, data completeness, safety actions, and transition rates from temporary access to routine reimbursement.

## 6 Conclusion and outlook

Early and accelerated access programs for medical devices in the EU provide crucial pathways for patients to obtain innovative care in advance of full market integration. Our review highlighted that they have become important in light of devices in higher MDR risk classes (notably Classes IIb and III) and drug-device combination medical devices, which are subject to stringent regulatory approval pathways.

This review showed that, unlike medicinal products, the EU plays a smaller role than MSs in providing early access to medical devices. Nevertheless, through derogations provided in the EU-MDR, the EU has set the base for early and accelerated access programs in MSs. However, national applications differ strongly, reducing transparency and utilisation.

The policy landscape described in this review should be interpreted as a snapshot up to June 2025, while the EU medical device framework is evolving. In particular, on 16 December 2025 the European Commission published a proposal to amend the MDR and IVDR to simplify and reduce regulatory burden and to introduce a dedicated mechanism for designated *breakthrough* and *orphan* devices through a new Article 52a [8]. This development reinforces the case for *agile regulation* in areas where innovation dynamics and iterative software updates imply that evidence and risk profiles are not static, consistent with OECD guidance on agile regulatory governance [34].

## Footnotes

^1^In this paper, we use “breakthrough” in a descriptive (non-legal) sense aligned with MDCG 2025-9: a device is considered breakthrough if it (i) introduces a high degree of novelty with respect to the device technology, the related clinical procedure, and/or its application in clinical practice, and (ii) is reasonably expected to provide a significant positive clinical impact on patients or public health for a life-threatening or irreversibly debilitating disease or condition [32].

## Glossary

AAP: Accelerated Access Program(s)
AI: Artificial Intelligence
AIA: Artificial Intelligence Act [Regulation (EU) 2024/1689]
AIB: Artificial Intelligence Board
AIMDD: Active Implantable Medical Device Directive (90/385/EEC)
ANS: Agence du Numérique en Santé (French Digital Health Agency)
ANSM: Agence nationale de sécurité du médicament et des produits de santé (French National Agency for Medicines and Health Products Safety)
BeNeLuxA: Benelux–Austria Initiative for cross-country cooperation in health
BfArM: Bundesinstitut für Arzneimittel und Medizinprodukte (Federal Institute for Drugs and Medical Devices, Germany)
CE: Conformité Européenne (EU conformity marking)
CECP: Clinical Evaluation Consultation Procedure
CED: Coverage with Evidence Development
CEE: Central and Eastern Europe
CEPS: Comité économique des produits de santé (Economic Committee for Health Products)
CNAM: Caisse Nationale de l’Assurance Maladie (National Health Insurance Fund)
DiGA: Digitale Gesundheitsanwendung (Digital Health Application)
EAP: Early Access Program(s)
EC: European Commission
EEC: European Economic Community
EHR: Electronic Health Record(s)
EMA: European Medicines Agency
EU: European Union
EU-MDR: European Union Medical Device Regulation [Regulation (EU) 2017/745]
EUDAMED: European Database on Medical Devices
Eur-Lex: EU law and publications portal
FAMHP: Federal Agency for Medicines and Health Products (Belgium)
FDA: Food and Drug Administration (United States)
FI: Forfait Innovation (French innovation funding scheme)
GCP: Good Clinical Practice
GSPR: General Safety and Performance Requirement(s)
HAS: Haute Autorité de Santé (French National Authority for Health)
HTA: Health Technology Assessment
HUD: Humanitarian Use Device
ICD-10: International Classification of Diseases, 10th Revision
IMDRF: International Medical Device Regulators Forum
ISO: International Organization for Standardization
IVDR: In Vitro Diagnostic Medical Devices Regulation [Regulation (EU) 2017/746]
JAZMP: Agency for Medicinal Products and Medical Devices of Slovenia
KPI: Key Performance Indicator
LOINC: Logical Observation Identifiers Names and Codes
MDCE: Medical Device Clinical Evaluation
MDCG: Medical Device Coordination Group
MDD: Medical Devices Directive (93/42/EEC)
MDR: Medical Devices Regulation [Regulation (EU) 2017/745]
MDSW: Medical Device Software
MEA: Managed Entry Agreement
MedDRA: Medical Dictionary for Regulatory Activities
MS: Member State
NUB: Neue Untersuchungs- und Behandlungsmethoden (new examination and treatment methods, Germany)
OECD: Organisation for Economic Co-operation and Development
PECAN: Prise en charge anticipée numérique (Digital Advance Coverage)
PECT: Prise En Charge Transitoire (French transitional coverage)
PMCF: Post-Market Clinical Follow-up
PMS: Post-Market Surveillance
PPE: Personal Protective Equipment
PRISMA-ScR: Preferred Reporting Items for Systematic reviews and Meta-Analyses – Scoping Review
QALY: Quality-Adjusted Life Year
RWD: Real-World Data
RWE: Real-World Evidence
SaMD: Software as a Medical Device
SGB: V Sozialgesetzbuch Fünftes Buch (German Social Code, Book V)
SNOMED: CT Systematized Nomenclature of Medicine—Clinical Terms
UDI: Unique Device Identification
UDI-DI: Unique Device Identification—Device Identifier
UDI-PI: Unique Device Identification—Production Identifier
US: United States
