# Supplementary material for: Early and accelerated access programs for medical devices in the European Union: mapping regulatory derogations and national schemes
Source: Front Med Technol. 2026 Feb 13;8:1729631. doi: 10.3389/fmedt.2026.1729631 (PMC12946103; doi:10.3389/fmedt.2026.1729631)
Supplement: Supplementary file 1 [file Table2.pdf]

Table 2: Overview of EU-level legal and guidance documents relevant to early and accelerated access for medical devices (EU framework).

Document	Type	Year	Main scope	Relevance for early/accelerated access (key points)
Regulation (EU) 2017/745 on medical devices (MDR) [15]	EU Regulation (binding law)	2017 (applicable 2021)	Medical devices (placing on the market / putting into service; conformity assessment; PMS/PMCF)	Defines CE-marking baseline; provides legal bases for derogations and non-CE routes relevant to early use (notably Art. 59), plus specific regimes (custom-made; Art. 5(5) health institution manufacture) that can enable patient access outside routine CE pathways.
Regulation (EU) 2017/746 on in vitro diagnostic medical devices (IVDR) [14]	EU Regulation (binding law)	2017 (phased application)	In vitro diagnostic medical devices	Analogous baseline for IVDs; relevant where early access mechanisms apply to IVDs and for the interpretation of software/AI IVD tools; interacts with derogations and national practices.

Continued on next page

Document	Type	Year	Main scope	Relevance for early/accelerated access (key points)
MDR Article 59 (within Regulation (EU) 2017/745) [15]	EU legal provision (binding)	2017	National derogations from conformity assessment / CE-marking requirements	Core EU-level legal "emergency valve" enabling Member State competent authorities to authorise placing on the market / putting into service of specific non-CE devices in the interest of public health or patient safety/health; includes notification duties and possible EU-level assessment/extension.
MDR Article 5(5) (health institution in-house manufacture) [15]	EU legal provision (binding)	2017	In-house manufactured devices used within health institutions	Creates a non-CE route under strict conditions (QMS/documentation/justification of unmet needs vs equivalent CE device); relevant to early access in hospital settings and to evidence-generation obligations linked to PMS/PMCF.

Continued on next page

Document	Type	Year	Main scope	Relevance for early/accelerated access (key points)
MDR provisions on custom-made devices (incl. definition and obligations) [15]	EU legal provisions (binding)	2017	Custom-made devices	Permits supply without CE-marking for individually prescribed devices, subject to documentation and applicable safety/performance requirements; relevant as an alternative early-use channel distinct from Art. 59 derogations.
European Commission guidance on COVID-19 related derogations / facilitation measures (incl. use of Art. 59 in crisis) [7]	EC guidance (non-binding)	2020	Crisis use of derogations / market surveillance and emergency facilitation	Illustrates how Art. 59 and national emergency measures were operationalised in a public health crisis; useful as an applied example of expedited derogations (but context-specific).
MDCG 2019-11 (rev. 1, June 2025): Qualification and classification of software under MDR/IVDR [30]	MDCG guidance (non-binding, influential)	2019 (rev. 2025)	Medical device software (MDSW); Rule 11 classification logic	Supports consistent EU interpretation of when software is a medical device and how it is risk-classified (often IIa/IIb/III); relevant to determining which software products fall into higher-risk segments where early/accelerated access is most salient.

Continued on next page

Document	Type	Year	Main scope	Relevance for early/accelerated access (key points)
MDCG 2020-1: Clinical evaluation / clinical evidence for medical device software [28]	MDCG guidance (non-binding, influential)	2020	Clinical evidence expectations for MDSW	Clarifies evidence expectations for software, shaping feasibility of accelerated pathways and "learning-by-using" obligations (especially for iterative updates and lifecycle evidence).
MDCG 2025-6 (June 2025): FAQ on interplay between MDR/IVDR and the AI Act (with aib) [31]	MDCG/aib guidance (non-binding)	2025	Regulatory interface MDR/IVDR + AI Act	Clarifies dual compliance expectations for AI-enabled devices; relevant to accelerated access because it affects conformity assessment scope, post-market obligations, and governance of updates (change control / monitoring).
MDCG 2025-9: Guidance on Breakthrough Devices (BtX) under MDR/IVDR [32]	MDCG guidance (non-binding, influential)	2025	Breakthrough device framing in EU context	EU-specific reference for "major clinical benefit vs state of the art" and "unmet medical need" framing; supports consistent EU context when discussing breakthrough-type acceleration and prioritisation concepts.

Continued on next page

**Document**	**Type**	**Year**	**Main scope**	**Relevance for early/accelerated access (key points)**
European Commission proposal COM(2025) 1023 final (and annex/ACT reference) [8**?**]	Legislative proposal (non-binding until adopted)	2025	Proposed amendments to MDR/IVDR (incl. acceleration tools)	Signals prospective EU-level acceleration tools (e.g., proposed Article 52a modalities for designated breakthrough/orphan devices); relevant for "outlook" and policy trajectory rather than current law.
