# Supplementary material for: Early and accelerated access programs for medical devices in the European Union: mapping regulatory derogations and national schemes
Source: Front Med Technol. 2026 Feb 13;8:1729631. doi: 10.3389/fmedt.2026.1729631 (PMC12946103; doi:10.3389/fmedt.2026.1729631)
Supplement: Supplementary file 2 [file Table3.pdf]

Table 3: EU-27 overview of mechanisms for early access (**EAP**), emergency/public-health **derogations**, and accelerated/conditional payer access (**AAP**) for medical devices, with publicly documented examples where available.

Reading note: Many Member States implement exceptional pre-CE access primarily via MDR Article 59 / IVDR Article 54. Public disclosure of granted derogations and named device examples is uneven.

Evidence strength: A = official public source(s) + named device example; B = official public source(s) but no named device example; C = not readily documented nationally (relies on EU framework/contact points).

**Member State**	**EAP (exceptional pre-CE use)**	**Derogation (emergency / public health)**	**AAP (accelerated coverage / CED / fast track)**	**Example device that benefited (publicly documented)**	**Evidence**
Austria	Competent authority (BASG): time-limited authorisation possible under MDR Art. 59 / IVDR Art. 54 (national implementation)	Same legal basis (Art. 59/54) for urgent public-health interest; case-by-case BASG handling	No consistently documented national device CED/fast-track scheme; coverage via standard assessment/procurement	No publicly named device located (device-by-device derogation decisions not routinely published)	B

Continued on next page

Member State	EAP (exceptional pre-CE use)	Derogation (emergency / public health)	AAP (accelerated coverage / CED / fast track)	Example device that benefited (publicly documented)	Evidence
Belgium	FAMHP "exceptional use" / derogation request under MDR Art. 59 / IVDR Art. 54 (public forms/process)	Same mechanism for emergency/public-health interest; device-specific decisions not systematically published as a central list	INAMI/RIZIV conditional reimbursement mechanisms for devices (e.g., limited clinical application cohorts; implant evaluation/listing processes)	Implantable CGM limited clinical application (published cohort/codes); TAVI reimbursement coding updates referenced in published nomenclature/regulatory documents	A
Bulgaria	Competent authority: Bulgarian Drug Agency (BDA). National derogations possible under MDR Art. 59 / IVDR Art. 54; device lists not routinely public	Same mechanism for exceptional public-health need; handled case-by-case	No nationally branded device CED/fast-track scheme consistently documented in public sources	No publicly named device located	C

Continued on next page

**Member State**	**EAP (exceptional pre-CE use)**	**Derogation (emergency / public health)**	**AAP (accelerated coverage / CED / fast track)**	**Example device that benefited (publicly documented)**	**Evidence**
Croatia	Competent authority: HALMED. National derogations possible under MDR Art. 59 / IVDR Art. 54; device lists not routinely public	Same mechanism for public-health interest; handled case-by-case	No clearly documented national device CED/fast-track scheme in public sources	No publicly named device located	C
Cyprus	Competent authority: CymDA. National derogations possible under MDR Art. 59 / IVDR Art. 54; no consolidated, device-specific public list located	Same mechanism for emergency/public health; handled case-by-case	No clearly documented national device CED/fast-track scheme in public sources	No publicly named device located	C

Continued on next page

**Member State**	**EAP (exceptional pre-CE use)**	**Derogation (emergency / public health)**	**AAP (accelerated coverage / CED / fast track)**	**Example device that benefited (publicly documented)**	**Evidence**
Czech Republic	Competent authority: SÚKL. National exemption permission for placing on market/putting into service aligned with MDR Art. 59 / IVDR Art. 54	Same mechanism for urgent need/public-health interest; handled by SÚKL on justified request	Reimbursement mainly via standard categorisation/coverage processes; no single branded national CED program for devices found	No publicly named device located (public sources focus on process rather than listing named derogated devices)	B
Denmark	Competent authority: Danish Medicines Agency. Public process exists for "Derogation from CE marking" (Art. 59/54 logic)	Same mechanism for emergency/public health interest; handled case-by-case	No clearly documented national device CED/fast-track scheme in public sources	No publicly named device located	B

Continued on next page

Member State	EAP (exceptional pre-CE use)	Derogation (emergency / public health)	AAP (accelerated coverage / CED / fast track)	Example device that benefited (publicly documented)	Evidence
Estonia	Competent authority: Ravimiamet. Public information on exceptions allowing time-limited placing on market/putting into service for non-compliant devices (Art. 59/54-type logic)	Same mechanism for urgent public-health interest; handled case-by-case	No clearly documented national device CED/fast-track scheme in public sources	No publicly named device located	B
Finland	Competent authority: Fimea. Public "exemption permit" process explicitly references MDR Art. 59 / IVDR Art. 54 (time-limited)	Same legal pathway for urgent/public-health needs; handled case-by-case	No clearly documented national device CED/fast-track scheme in public sources	No publicly named device located	B

Continued on next page

Member State	EAP (exceptional pre-CE use)	Derogation (emergency / public health)	AAP (accelerated coverage / CED / fast track)	Example device that benefited (publicly documented)	Evidence
France	ANSM publishes a detailed MDR Art. 59 derogation procedure and applicant guidance (incl. online process)	Same mechanism supports urgent/public-health derogations; communications may be category-based rather than exhaustive device lists	Structured accelerated/conditional coverage exists for devices: Forfait Innovation, PECT (transitional coverage), PECAN (digital/remote monitoring)	Neovasc Reducer (publicly announced as first PECT device); Named devices in official orders/coverage decisions (e.g., SONOCLOUD-9, ENDOTEST)	A
Germany	BfArM: national derogation / "Sonderzulassung" possible under MDR Art. 59 / IVDR Art. 54 (guidance public; granted cases not always listed centrally)	Same mechanism for public-health interest/emergency; handled case-by-case	DiGA Fast-Track (official directory with provisional/final listing; evidence generation possible during provisional listing)	Named DiGA products are published in the official DiGA directory (publicly accessible, updated)	A

Continued on next page

Member State	EAP (exceptional pre-CE use)	Derogation (emergency / public health)	AAP (accelerated coverage / CED / fast track)	Example device that benefited (publicly documented)	Evidence
Greece	Competent authority: EOF. National derogations possible under MDR Art. 59 / IVDR Art. 54; device lists not routinely public	Same mechanism for emergency/public health; case-by-case	No clearly documented national device CED/fast-track scheme in public sources	No publicly named device located	C
Hungary	National deviation permission exists aligned with MDR Art. 59 (public legal text/description available in Hungarian)	Same mechanism for public-health interest/emergency under deviation permission rules	No clearly documented national device CED/fast-track scheme in public sources	No publicly named device located	B

Continued on next page

Member State	EAP (exceptional pre-CE use)	Derogation (emergency / public health)	AAP (accelerated coverage / CED / fast track)	Example device that benefited (publicly documented)	Evidence
Ireland	HPRA public MDR transitional provisions reference national derogations under Art. 59 (and Art. 97) with case-by-case handling	Same mechanism for urgent/public-health interest; handled case-by-case	No clearly documented national device CED/fast-track scheme in public sources	No publicly named device located	B
Italy	Competent authority: Ministry of Health. National derogations possible under MDR Art. 59 / IVDR Art. 54; public circulars/process references exist but device lists not routinely published	Same mechanism for public-health interest/emergency; case-by-case under ministry process	No single national device CED/fast-track scheme consistently documented; coverage often regional/procurement-driven	No publicly named device located	B

Continued on next page

**Member State**	**EAP (exceptional pre-CE use)**	**Derogation (emergency / public health)**	**AAP (accelerated coverage / CED / fast track)**	**Example device that benefited (publicly documented)**	**Evidence**
Latvia	Competent authority: national CA listed in EU contact points; national derogations possible under MDR Art. 59 / IVDR Art. 54 but no easily findable consolidated national public guidance located	Same mechanism for public-health interest; case-by-case	No clearly documented national device CED/fast-track scheme in public sources	No publicly named device located	C

Continued on next page

Member State	EAP (exceptional pre-CE use)	Derogation (emergency / public health)	AAP (accelerated coverage / CED / fast track)	Example device that benefited (publicly documented)	Evidence
Lithuania	Competent authority: national CA listed in EU contact points; national derogations possible under MDR Art. 59 / IVDR Art. 54 but no easily findable consolidated national public guidance located	Same mechanism for public-health interest; case-by-case	No clearly documented national device CED/fast-track scheme in public sources	No publicly named device located	C
Luxembourg	Competent authority: Ministry of Health. Public derogation request form exists (process described; no device list)	Same mechanism for public-health interest; case-by-case	No clearly documented national device CED/fast-track scheme in public sources	No publicly named device located	B

Continued on next page

**Member State**	**EAP (exceptional pre-CE use)**	**Derogation (emergency / public health)**	**AAP (accelerated coverage / CED / fast track)**	**Example device that benefited (publicly documented)**	**Evidence**
Malta	Competent authority: Medicines Authority. Public derogation application form exists referencing MDR Art. 59 / IVDR Art. 54	Same mechanism for public-health interest; case-by-case	No clearly documented national device CED/fast-track scheme in public sources	No publicly named device located	B
Netherlands	Competent authority/oversight: IGJ and national bodies. National derogations possible under MDR Art. 59 / IVDR Art. 54; device lists not routinely public	Same mechanism for urgent/public-health interest; case-by-case	Vergoeding in Onderzoek (ViO): conditional reimbursement within research; (historically) conditional admission instruments preceded ViO	TAVI reimbursement scope/position documented in public package/position decisions	A

Continued on next page

**Member State**	**EAP (exceptional pre-CE use)**	**Derogation (emergency / public health)**	**AAP (accelerated coverage / CED / fast track)**	**Example device that benefited (publicly documented)**	**Evidence**
Poland	Competent authority: URPL. National derogations possible under MDR Art. 59 / IVDR Art. 54; consolidated public guidance varies; device lists not routinely public	Same mechanism for emergency/public health; case-by-case	No clearly documented national device CED/fast-track scheme in public sources	No publicly named device located	C
Portugal	Competent authority: INFARMED. National legal framework references Art. 59-type exceptional authorisation; device lists not routinely public	Same mechanism for public-health interest; case-by-case	No clearly documented national device CED/fast-track scheme in public sources	No publicly named device located	B

Continued on next page

Member State	EAP (exceptional pre-CE use)	Derogation (emergency / public health)	AAP (accelerated coverage / CED / fast track)	Example device that benefited (publicly documented)	Evidence
Romania	Competent authority: ANMDMR. Public communication explicitly describes derogation/authorisation under MDR Art. 59 (exceptional, time-limited)	Same mechanism for public-health interest/emergency described publicly	No clearly documented national device CED/fast-track scheme in public sources	No publicly named device located	B
Slovakia	Competent authority: ŠÚKL. National derogations possible under MDR Art. 59 / IVDR Art. 54; no easily findable consolidated public guidance located	Same mechanism for urgent/public-health interest; case-by-case	No clearly documented national device CED/fast-track scheme in public sources	No publicly named device located	C

Continued on next page

**Member State**	**EAP (exceptional pre-CE use)**	**Derogation (emergency / public health)**	**AAP (accelerated coverage / CED / fast track)**	**Example device that benefited (publicly documented)**	**Evidence**
Slovenia	Competent authority: JAZMP. National derogations possible under MDR Art. 59 / IVDR Art. 54; no easily findable consolidated public guidance located	Same mechanism for urgent/public-health interest; case-by-case	No clearly documented national device CED/fast-track scheme in public sources	No publicly named device located	C
Spain	Competent authority: AEMPS. Public guidance documents reference MDR Art. 59 derogations in transitional/market-access context	Same mechanism for public-health interest/emergency; case-by-case (no public device list)	No uniform national device fast-track/CED scheme across all regions; coverage often varies by Autonomous Community	No publicly named device located	B

Continued on next page

Member State	EAP (exceptional pre-CE use)	Derogation (emergency / public health)	AAP (accelerated coverage / CED / fast track)	Example device that benefited (publicly documented)	Evidence
Sweden	Competent authority: Läkemedelsverket. Public application page exists for MDR Art. 59/IVDR Art. 54 derogation (process described; no device list)	Same mechanism for public-health interest; case-by-case	No clearly documented national device CED/fast-track scheme in public sources (device coverage often regional)	No publicly named device located	B
